# Perceptions and Needs of Artificial Intelligence in Health Care to Increase Adoption: Scoping Review

**DOI:** 10.2196/32939

**Published:** 2022-01-14

**Authors:** Han Shi Jocelyn Chew, Palakorn Achananuparp

**Affiliations:** 1 Alice Lee Centre for Nursing Studies Yong Loo Lin School of Medicine National University of Singapore Singapore Singapore; 2 Living Analytics Research Centre Singapore Management University Singapore Singapore

**Keywords:** artificial intelligence, health care, service delivery, perceptions, needs, scoping, review

## Abstract

**Background:**

Artificial intelligence (AI) has the potential to improve the efficiency and effectiveness of health care service delivery. However, the perceptions and needs of such systems remain elusive, hindering efforts to promote AI adoption in health care.

**Objective:**

This study aims to provide an overview of the perceptions and needs of AI to increase its adoption in health care.

**Methods:**

A systematic scoping review was conducted according to the 5-stage framework by Arksey and O’Malley. Articles that described the perceptions and needs of AI in health care were searched across nine databases: ACM Library, CINAHL, Cochrane Central, Embase, IEEE Xplore, PsycINFO, PubMed, Scopus, and Web of Science for studies that were published from inception until June 21, 2021. Articles that were not specific to AI, not research studies, and not written in English were omitted.

**Results:**

Of the 3666 articles retrieved, 26 (0.71%) were eligible and included in this review. The mean age of the participants ranged from 30 to 72.6 years, the proportion of men ranged from 0% to 73.4%, and the sample sizes for primary studies ranged from 11 to 2780. The perceptions and needs of various populations in the use of AI were identified for general, primary, and community health care; chronic diseases self-management and self-diagnosis; mental health; and diagnostic procedures. The use of AI was perceived to be positive because of its availability, ease of use, and potential to improve efficiency and reduce the cost of health care service delivery. However, concerns were raised regarding the lack of trust in data privacy, patient safety, technological maturity, and the possibility of full automation. Suggestions for improving the adoption of AI in health care were highlighted: enhancing personalization and customizability; enhancing empathy and personification of AI-enabled chatbots and avatars; enhancing user experience, design, and interconnectedness with other devices; and educating the public on AI capabilities. Several corresponding mitigation strategies were also identified in this study.

**Conclusions:**

The perceptions and needs of AI in its use in health care are crucial in improving its adoption by various stakeholders. Future studies and implementations should consider the points highlighted in this study to enhance the acceptability and adoption of AI in health care. This would facilitate an increase in the effectiveness and efficiency of health care service delivery to improve patient outcomes and satisfaction.

## Introduction

### Background

Rapid advances in artificial intelligence (AI)—software systems designed to mimic human intelligence or cognitive functions—have sparked confidence in its potential to enhance the efficiency of health care service delivery and patient outcomes [1-3]. However, although AI has been rapidly adopted in many industries, such as finance and information technology (IT), its adoption in health care remains relatively lagged because of the ethical and safety considerations that are more pronounced when it comes to human lives at stake [4]. AI-powered systems in health care can autonomously or semiautonomously perform a wide variety of tasks, such as medical diagnosis [5], treatment [6], and self-monitoring and coaching [7,8]. In some studies, AI has been shown to outperform human capabilities, such as analyses of chest x-ray images by radiologists [9]. Not only is AI expected to improve the quality of care and health outcomes for patients by decreasing human errors, but it is also likely to free up time for clinicians and health care workers from routine and repetitive tasks, enabling them to focus on more complex tasks [9,10]. For instance, in many areas of medical imaging, the use of fast and accurate AI-assisted diagnoses would significantly increase the workflow efficiency by processing more than 250 million images per day [11]. Various AI chatbots have also been developed to provide mental health counseling and assist overburdened clinicians [9]. Through AI-enabled apps and wearable devices, patients and the public could self-monitor and self-diagnose symptoms, such as atrial fibrillation, skin lesions, and retinal diseases [9].

Owing to the emerging nature of modern AI systems, the perceptions and needs of affected stakeholders (eg, health care providers, patients, caregivers, policy makers, and IT technicians) on the use of AI in health care are not yet fully understood. A large body of literature suggests that human factors, such as trust, perceived usefulness, and privacy, play an important role in the acceptance and adoption of past technologies in health care, including handheld devices [12], IT [13], and assistive technologies [14]. However, current evidence remains broad and general, and little is known about the perceptions and needs of AI in community health care. As the world makes a paradigm shift from curative to preventive medicine, AI holds a strong transformative potential to enhance sustainable health care by empowering self-care, such as self-monitoring and self-diagnosis. However, it is important to first understand the perspectives of all direct users of AI-driven systems (eg, patients and frontline health workers) and their perceived needs to ensure its successful adoption across different parts of the health care sector, especially community health care. Thus, this study aims to present an overview of the perceptions and needs of AI in community health care. The implications of this study will help inform the design of future health care–related AI technology to better fit the needs of users and enhance the adoption and acceptability of the technology.

### Definition of AI

First, as the term *AI* is broadly used in many disciplines to represent various forms of intelligent systems and algorithms, it is important to establish a concrete and unified definition of AI for this study. Specifically, we adopted the definition of AI proposed by the High-Level Expert Group on Artificial Intelligence [15], which describes AI in terms of both a technology and field of study:

Artificial intelligence (AI) systems are software (and possibly also hardware) systems designed by humans that, given a complex goal, act in the physical or digital dimension by perceiving their environment through data acquisition, interpreting the collected structured or unstructured data, reasoning on the knowledge, or processing the information, derived from this data and deciding the best action(s) to take to achieve the given goal. AI systems can either use symbolic rules or learn a numeric model, and they can also adapt their behaviour by analysing how the environment is affected by their previous actions.

As a scientific discipline, AI includes several approaches and techniques, such as machine learning (of which deep learning and reinforcement learning are specific examples), machine reasoning (which includes planning, scheduling, knowledge representation and reasoning, search, and optimization), and robotics which includes control, perception, sensors, and actuators, as well as the integration of all other techniques into cyber-physical systems.

Furthermore, most, if not all, modern AI systems are considered artificial narrow intelligence (ANI) or *Weak AI* [15] designed to perform one or more specific tasks. In health care, domain-specific tasks for ANI may vary from performing human perceptions, such as image recognition [16] and natural language processing [17], to making complex clinical decisions, such as medical diagnostics [18]. Many recent advances and breakthroughs in ANI use learning-based approaches, namely, deep learning, in which computational models consisting of several layers of artificial neural networks (hence the titular *deep*) are trained by learning from a massive amount of sample data to perform specific tasks. Although recent performances of ANI appear very promising, ANI models are limited in their generalizability, that is, models trained to perform tasks in one domain cannot be generalized to other domains. For example, ANI trained to diagnose diabetic retinopathy from fundus images cannot be directly used to detect pneumonia from chest x-ray images. In contrast to ANI, artificial general intelligence (AGI) or *Strong AI* [15] belongs to a class of AI that displays true human intelligence, capable of continuously learning and performing any tasks like a real human. AGI is most likely in public consciousness when talking about AI, as it is frequently portrayed in popular culture by sentient robots and self-aware systems. At present, no AI systems have been able to come close to exhibit the AGI capability. For a useful and concise summary regarding the definitions, terminologies, and history of AI, see the following technical reports: Ethics Guidelines for Trustworthy AI [15] and Historical Evolution of Artificial Intelligence [19].

## Methods

A systematic scoping review was conducted according to the 5-stage framework by Arksey and O’Malley [20]. Results were reported according to the PRISMA (Preferred Reporting Items for Systematic Reviews and Meta-Analyses) checklist (Multimedia Appendix 1) [21].

### Stage 1: Identifying the Research Question

Our research question was as follows: What is known about the perceptions and needs of AI in health care?

### Stage 2: Identifying Relevant Studies

Studies were searched from inception until June 21, 2021, using a 3-step search strategy. First, potential keywords and Medical Subject Headings terms were generated through iterative searches on PubMed and Embase. Keywords such as machine learning did not result in better search outcomes (ie, many irrelevant results were retrieved, such as the use of machine learning to explore perceptions of other topics); hence, they were omitted. Next, keywords including *artificial intelligence, AI*; *public*; *consumer*; *community*; perception*; preference*; needs*; opinions*; and *acceptability* were searched through nine databases: ACM Library, CINAHL, Cochrane Central, Embase, IEEE Xplore, PsycINFO, PubMed, Scopus, and Web of Science. Additional articles were also retrieved from the first 10 pages of the Google Scholar search results and the reference lists of the included full-text articles. The specific database searches combined with Boolean operators are detailed in Multimedia Appendix 2.

### Stage 3: Study Selection

After removing duplicate articles, titles and abstracts were first screened by HSJC for inclusion eligibility. Articles were included if they were (1) focused on the use of AI in health care, except those focused on using AI to improve surgical techniques; (2) focused on perceptions, needs, and acceptability of AI in health care; (3) empirical studies or systematic reviews; (4) on adults aged ≥18 years; and (5) used in a community setting. Articles were excluded if they were (1) not specific to AI (eg, general eHealth or mobile health); (2) pilot studies, commentaries, perspectives, or opinion papers; and (3) not presented in the English language. In total, 43 full-text articles were screened independently by both coauthors, and discrepancies were resolved through discussions and consensus.

### Stage 4: Charting the Data

Data were extracted by HSJC using Microsoft Excel according to the following headings: author, year, title, aim, type of publication, study design, country, AI applications in health care, data collection method, population characteristics, sample size, age (mean or range), proportion of men, acceptability, perceptions, needs and preferences, and limitations.

## Results

### Stage 5: Collating, Summarizing, and Reporting Results

**Figure 1:**
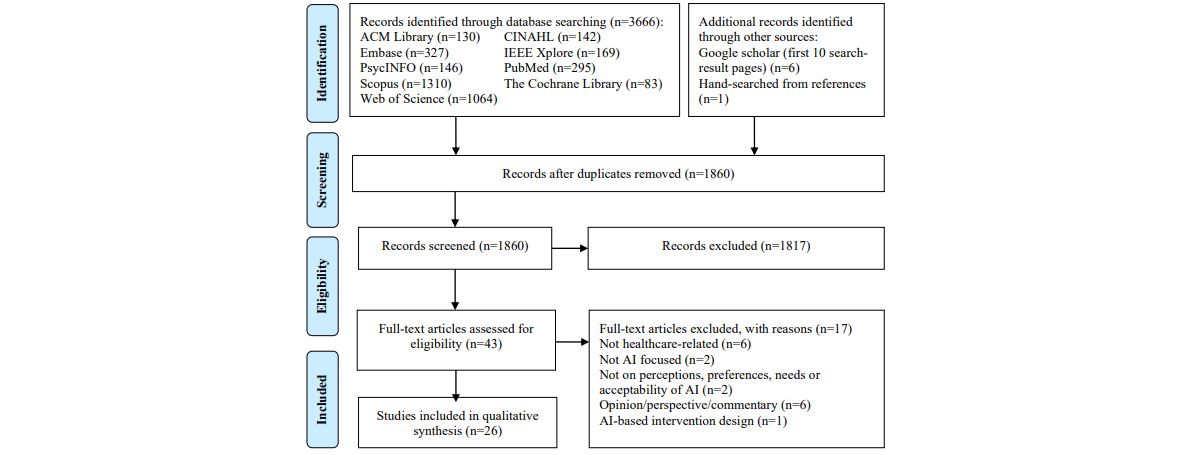
PRISMA (Preferred Reporting Item for Systematic Reviews and Meta-Analyses) flow diagram of search strategy. AI: artificial intelligence.

A total of 3666 articles were retrieved from the initial search. After removing duplicate articles, 50.74% (1860/3666) of titles and abstracts were screened, and 0.91% (17/1860) of full-text articles were excluded for reasons shown in Figure 1. A total of 1.4% (26/1860) of articles were included in this study, with the study characteristics summarized in Table 1 and detailed in Multimedia Appendix 3 [22-47]. The mean age of participants ranged from 30 to 72.6 years, and the proportion of men ranged from 0% to 73.4%. Sample sizes for studies with human subject responses ranged from 11 to 2780, and secondary data (ie, journal articles and app reviews) ranged from 31 to 1826 [22-24]. Interestingly, 19% (5/26) of studies focused on the use of chatbots in health care [23-27] and 31% (8/26) of studies measured acceptability using questionnaires, surveys, interviews [25,26,28-33], and the discrete choice experiment (Multimedia Appendix 4 [22-32,34,36,37,39,41-44,47]) [34]. All the studies showed at least moderate acceptability, or >50% of the participants showed acceptance toward the use of AI in health care, albeit only for minor conditions [26]. Age, IT skills, preference for talking to computers, perceived utility, positive attitude, and perceived trustworthiness were found to be associated with AI acceptability [25,26].

**Table 1:** Summary of study characteristics (N=26).

Study characteristics		Value, n (%)
**Country**		
	Australia and New Zealand [35]	1 (4)
	Canada [27,36-38]	4 (15)
	China [22,32,33,39,40]	6 (23)
	France [41]	1 (4)
	India [24,42]	2 (8)
	Korea [48]	1 (4)
	Saudi Arabia [29]	1 (4)
	Switzerland [30]	1 (4)
	United Kingdom [23,26,31,43,44]	5 (19)
	United Kingdom, Cyprus, Australia, the Netherlands, Sweden, Spain, United States, and Canada [28]	1 (4)
	United States [25,45,46]	3 (12)
**Type of publication**		
	Journal papers [22-29,31-41,43-47]	24 (92)
	Conference papers [30,42]	2 (8)
**Study design**		
	Observational [22,24,27-30,33-35,39,43-47]	15 (58)
	Qualitative [36-38,41,42]	5 (19)
	Mixed methods [25,26,31,32,40]	5 (19)
	Systematic review [23]	1 (4)
**Population characteristics**		
	General public [22,24,26,30,32-34,37,45]	9 (35)
	Health care, government, technology, and industrial staff [27-29,35,36,40-44]	10 (39)
	Patients and caregivers with specific diseases [25,31,34,36,38,39,47]	7 (27)
	Mixture (systematic review) [23]	1 (4)
**Artificial intelligence applications in health care**		
	General health care [22,23,26,27,29,33,36,37,40,41,43]	11 (42)
	Primary [44] and community health care [28,42]	3 (12)
	Chronic disease self-management [25,31,47]	3 (12)
	Self-diagnosis [30,32,34,39]	4 (15)
	Mental health [24,38]	2 (8)
	Diagnostics [35,45,46]	3 (12)

### Positive Perceptions

#### Overview

Several positive perceptions on the use of AI in health care were highlighted in our findings (Table 2).

**Table 2:** Perceptions on the use of artificial intelligence (AI) in health care.

Study	Available on demand and user-friendly	Efficiency	Price	Lack of trust in data privacy	Lack of trust in patient safety	Lack of trust in technology	Concerns over full automation
Abdi et al [28]	Able to collect data nonintrusively	Could support the self-care needs of older people—mobility, self-care and domestic life, social life and relationships, psychological support, and access to health care; potential uses for remote monitoring and prompting daily reminders, for example, medications	Cost was seen as both a facilitator of and a barrier to the older people’s adoption of AI^a^	Especially in voice-activated devices	Deemed technically and commercially ready to support the care needs of older people	NS^b^	NS
Abdullah and Fakieh [29]	NS	Speeds up health care processes	NS	NS	AI was unable to provide opinions in unexpected situations	NS	Most health care employees feared that the AI would replace their job (mean score 3.11 of 4)
Baldauf et al [30]	Constant availability, not restricted by physical location	Quicker diagnosis and no waiting time	AI could be a cost-saving alternative	There were concerns over data privacy	Users were unsure about the legality of official medical certification and app trustworthiness	NS	Only a minority would rely solely on an AI-driven app for assessing health
Castagno and Khalifa [43]	NS	In all, 79% of health care staff believed AI could be useful or extremely useful in their field of work	NS	In all, 80% of health care staff believed there may be serious privacy issues	NS	NS	Overall, 10% of health care staff worried AI will replace their job
Easton et al [31]	NS	NS	NS	Patients were not concerned over data sharing	Patients were unsure whether to treat a chatbot as a real physician or an adviser	NS	NS
Gao et al [22]	NS	NS	NS	Distrust of AI companies accounted for a quarter of all negative opinions among social media users	Social media users were pessimistic about the immaturity of AI technology	NS	Less than half of the social media posts expressed that AI would completely or partially replace human doctors
Griffin et al [25]	NS	The majority were interested in using a chatbot to help manage medications, refills, communicate with care teams, and accountability toward self-care tasks	NS	There were concerns with chatbots providing too much information and invading privacy	There were concerns with chatbots making overwhelming demands for lifestyle changes	NS	NS
Kim [47]	NS	NS	NS	NS	NS	NS	NS
Lai et al [41]	NS	NS	NS	There were legal difficulties to access individual health data; regulate use; strike balance between health, social justice, and freedom; and need to achieve confidentiality and respect for privacy	NS	NS	NS
Li et al [32]	NS	NS	NS	NS	AI may not understand complex emotional problems and give incurable diagnoses; and unsure whether doctors would accept the information provided by the AI	NS	NS
Liu et al [34]	NS	NS	NS	NS	Majority were confident that AI diagnosis methods would outperform human clinician diagnosis methods because of higher accuracy	NS	Majority preferred to receive combined diagnoses from both AI and human clinicians
Liu et al [39]	NS	NS	Acceptability depends on the expense of AI diagnosis compared with that of physicians	NS	Accuracy was deemed the most important attribute for AI uptake	NS	NS
Liyanage et al [44]	NS	Improves efficiency through decision support to improve primary health care processes and pattern recognition in imaging	NS	NS	There were concerns over the risk of medical errors, bias, and secondary effects of using AI (eg, insurance)	NS	AI technology is still not competent to replace human decision-making in clinical scenarios
McCradden et al [36]	NS	Potential for faster and more accurate analyses; ability to use more data	NS	There were concerns about privacy, commercial motives, and other risks and mixed views about explicit consent for research. Transparency is needed	It still requires human verification of computer-aided decisions	NS	Fear of losing human touch and skills from overreliance on machines
McCradden et al [37]	NS	Predictive modeling performed on primary care health data and business analytics for primary care provider. AI has the potential to improve managerial and clinical decisions and processes, and this would be facilitated by common data standards	NS	Nonconsented use of health data is acceptable with disclosure and transparency. Selling health data should be prohibited. Some privacy health outcomes trade-off is acceptable	A few patients and caregivers felt that allocation of health resources should be done via computerized output, and a majority stated that it was inappropriate to delegate such decisions to a computer	NS	NS
Milne-Ives et al [23]	Easy to learn and use	Speed up the process of service delivery and performance. Respondents appreciated reminders and assistance in forming routines, chatbot agents in facilitating learning, and agents in providing accountability (eg, regular check-ins, follow-ups). Multi-modal interactions (eg, voice, touch) were viewed positively	NS	NS	Unable to sufficiently encompass the real situational complexity. Electronic physician did not have the ability to go deep enough, provide access to other materials, or provide enough information	NS	NS
Nadarzynski et al [26]	Chatbots were perceived as a convenient tool that could facilitate the seeking of health information on the web	If free at the point of access, chatbots were seen as time-saving and useful platforms for triaging users to appropriate health care services	NS	Some participants were concerned about the ability of the chatbots to keep sensitive data secured and confidential. The level of anonymity offered by chatbots was viewed positively by several participants	Risk of harm from inaccurate or inadequate advice. Immature in performing a diagnosis but providing general health advice is acceptable	Uncertain about the quality, trustworthiness, and accuracy of the health information provided by chatbots	NS
Okolo et al [42]	NS	AI app would be able to perform some of the manual tasks and make the work of CHWs^c^ more efficient, and help CHWs and patients in decision-making processes	NS	NS	Concerned over AI failures or misdiagnoses. The AI app might serve to reinforce the expertise of CHWs, improve patients’ understanding of the diagnosis		AI would never completely replace health care workers because of the need for human interaction
Palanica et al [27]	NS	Many physicians believed that chatbots would be most beneficial for administrative tasks such as scheduling physician appointments, locating health clinics, or providing medication information	NS	NS	Chatbots could be a risk to patients if they self-diagnose too often and did not accurately understand the diagnoses	NS	Chatbots alone are not able to provide effective care for all patients because of limited knowledge of personal factors
Prakash and Das [24]	Always available at the touch of a button and user-friendly	NS	The price of mental health chatbots could be a decisive factor in places with a poor health insurance system	Data privacy is a major barrier that prevents the adoption of mental health chatbots	Chatbots may be useful in managing mental health conditions but not good enough for complex problems. May even be more harmful to vulnerable patients with poor advice	Doubtful about reliability and functionality	NS
Scheetz et al [35]	NS	The top three potential advantages are improved patient access to disease screening; improved diagnostic confidence; and enhanced efficiency, that is, reduced time spent by specialists on monotonous tasks	NS	There were concerns over the divestment of health care to large technology and data companies	There were concerns over medical liability because of machine errors	AI would need to perform much more superior to the average specialist in screening and diagnosis	There is decreasing reliance on medical specialists for diagnosis and treatment advice
Stai et al [45]	NS	NS	Almost all (94%) participants were willing to pay for a review of medical imaging by an AI	NS	NS	Nearly equal trust in AI vs physician diagnoses; significantly more likely to trust an AI diagnosis of cancer over a physician’s diagnosis	NS
Sun and Medaglia [40]	NS	NS	High treatment costs for patients but does not make profits for hospitals	Lack of trust toward AI-based decisions; unethical use of shared data	Doubts in the ability of AI to identify country-specific patient disease profiles	There were concerns over the lack of data integration; standards of data collection, format, and quality; algorithm opacity; and ability to read unstructured data	NS
Tam-Seto et al [38]	It could support those not currently accessing mental health services	It would address the perceived mental health service gap	NS	No assurance of users’ privacy	Trust in the app, as it discloses that the app was informed by the Canadian military experience (credibility)	There were doubts over overall sustainability	NS
Xiang et al [33]	NS	Health care workers prefer AI to alleviate daily repetitive work and improve outpatient guidance and consultation. The current auxiliary and partial substitution effects of AI are recognized by >90% of the public, and both groups have positive attitudes regarding AI development	NS	NS	Both health care and non–health care workers express more trust in real doctors than in AI	NS	A very small minority of health care and non–health care workers expect that full automation is likely to happen
Zhang et al [46]	NS	NS	NS	There were concerns about cybersecurity	NS	There were concerns about accuracy, reliability, quality, and trustworthiness of AI outputs, such as the predictions and recommended medical information	Supplementary service rather than a replacement of the professional health force is required for the AI to be particularly useful in helping patients to comprehend their physician’s diagnosis

^a^AI: artificial intelligence.

^b^NS: not specified.

^c^CHW: community health care worker.

#### Availability and Ease of Use

Of the 26 studies, 3 (12%) studies highlighted the advantage of AI being constantly available without restrictions such as physical location, time, and access to a structured treatment [24,30,38]; 3 (12%) other studies also mentioned the appreciation of respondents for how an AI system could collect data remotely in a nonintrusive and user-friendly manner [23,24,28]. These studies mostly represented the perceptions of consumers and health care providers [24,30,38] (Multimedia Appendix 3). Only 4% (1/26) of studies did not mention the population characteristics [24].

#### Improves Efficiency and Reduces the Cost of Health Care Service Delivery

In all, 58% (15/26) of studies highlighted the potential of AI to improve the efficiency of health care service delivery in terms of remote monitoring [28], providing health-related reminders [23,28], increasing the speed and accuracy of health care processes (eg, consultation wait time, triaging, diagnosis, and managing medication refills) [26,29,30,35-37,44], facilitating care team communications, improving care accountability (eg, regular check-ins and follow-ups for information gathering) [23], and taking over repetitive manual tasks (eg, scheduling, patient education, and vital signs monitoring) [27]. Some respondents also appreciated the use of AI to provide a second opinion to physicians’ diagnoses or evaluations [42,46]. Overall, 12% (3/26) of studies [24,34,45] discussed the potential cost-saving capacity of AI that influences AI acceptability, whereas 4% (1/26) mentioned that the provision of an AI service using IBM Watson caused patients to incur higher treatment costs that did not translate to profits for the hospital after factoring onboarding of the technology [40]. There was a good proportion of representation from the health care and IT staff (53.3%) [27-29,36,37,40,42,44] and those from the public, including patients (Multimedia Appendix 3). Only 4% (1/26) of the studies did not mention the population characteristics [24].

### Concerns and Mitigation Strategies

#### Overview

Our findings highlight several concerns (Table 2) and mitigation strategies (Table 3).

**Table 3:** Needs and mitigation strategies of artificial intelligence (AI) in health care.

Study	Need for transparency, credibility, and regulation	Lack of personalization and customizability	Perceived empathy and personification	Design, user experience, and interconnectedness with other devices	Educating the public on AI capabilities
Abdi et al [28]	NS^a^	NS	NS	Implementing user-led design principles could facilitate the acceptability and uptake of these technologies	NS
Abdullah and Fakieh [29]	NS	NS	NS	NS	Most respondents had a general lack of AI knowledge (mean score 2.95 from 4) and were unaware of the advantages and challenges of AI applications in health care
Baldauf et al [30]	Need guarantee of anonymized transmission and analysis of personal health data of users	Personalized explanation of analyses Disease information Treatment cost Recommending physician’s visit Alternative Therapies Prevention information Treatment companion Mental support Objectivity and independence	Lack of personal face-to-face contact with a human expert	NS	NS
Castagno and Khalifa [43]	NS	NS	NS	NS	NS
Easton et al [31]	Needed clarity on whether the chatbot was a physician or an adviser	The system should allow personalization	The chatbot should be enriched by the ability to detect emotion (distress, fatigue, and irritation) in speech and nonverbal cues to build a therapeutic relationship between the agent and the patient	Personification of the chatbot should be emotionally expressive. Multi-modal interactions and interconnectedness with other consumer devices were suggested	NS
Gao et al [22]	NS	NS	NS	NS	NS
Griffin et al [25]	NS	NS	NS	Some older adults described limited use of smartphone, given the small screen or inability to keep track of it	NS
Kim [47]	NS	NS	NS	NS	NS
Laï et al [41]	Need for app regulation to create a more permissive regulatory framework; achieve confidentiality and respect for privacy	NS	NS	NS	NS
Li et al [32]	Credibility of the intelligent self-diagnosis system can be improved through transparency (eg, showing accuracy scores). State if doctors would accept information provided by AI	AI systems may provide more specific, personalized information and advice	NS	NS	NS
Liu et al [34]	NS	NS	NS	NS	NS
Liu et al [39]	NS	NS	NS	NS	NS
Liyanage et al [44]	NS	NS	NS	NS	NS
McCradden et al [36]	Need for transparency on how and by whom their data were used	NS	NS	NS	NS
McCradden et al [37]	Need for transparency, disclosure, reparations, deidentification of data, and use within trusted institutions	NS	NS	NS	NS
Milne-Ives et al [23]	NS	Need more customization or availability of feature options (eg, preformatted or free-text options)	Need for greater interactivity or relational skills in conversational agents. Respondents liked that the agent had a personality and showed empathy, which improves personal connection. Others had difficulty in empathizing with the agent or reported disliking its limited conversation and responses	Interaction was too long, the use of nonverbal expressions by the avatar was not appealing, and there was a lack of clarity regarding the aim of the chatbot. Better integration of the agent with electronic health record systems (for a virtual physician) or health care providers (for an asthma self-management chatbot) would be useful	NS
Nadarzynski et al [26]	Need to increase transparency of information source	NS	Lack of empathy and inability of chatbots to understand more emotional issues, especially in mental health. The responses given by chatbots were seen as depersonalized, cold, and inhuman. They were perceived as inferior to physician consultation, although anonymity could facilitate the disclosure of more intimate or uncomfortable aspects to do with health	NS	There was a general lack of familiarity and understanding of health chatbots among participants
Okolo et al [42]	NS	NS	NS	NS	NS
Palanica et al [27]	NS	NS	Many physicians believed that chatbots cannot display human emotion	NS	NS
Prakash and Das [24]	NS	There were user input restrictions during chatbot conversations where the chatbot forced the users to respond to a list of choices	Mixed findings on perceived empathy. Some users perceived the chatbot to be warm and friendly, whereas others found it to be unsympathetic and rude Mixed findings on preference for a life-like chatbot—some felt it a little creepy and weird The nonjudgmental nature of chatbots is a strong motivator of adoption. It should respond spontaneously in a contingent, human-like manner	NS	NS
Scheetz et al [35]	NS	NS	NS	NS	A minority (13.8%) of the participants felt that the specialist training colleges were adequately prepared for the introduction of AI into clinical practice. Education was identified as a priority to prepare clinicians for the implementation of AI in health care
Stai et al [45]	NS	NS	NS	NS	NS
Sun and Medaglia [40]	NS	NS	NS	NS	Insufficient knowledge on values and advantages of AI technology; unrealistic expectations toward AI technology
Tam-Seto et al [38]	NS	NS	NS	NS	Managing the public’s expectations of the capabilities of such an app
Xiang et al [33]	NS	NS	NS	NS	More than 90% of health care workers expressed a willingness to devote time to learning about AI and participating in AI research
Zhang et al [46]	Majority of participants expressed the need to increase system transparency by explaining how the AI arrived at its conclusion	Need more personalized and actionable information AI should be enhanced with features that can help to recommend personalized questions to ask physicians	Concerns over lack of empathy	NS	NS

^a^NS: not specified.

#### Lack of Trust

##### Data Privacy

In all, 58% (15/26) of studies described the respondents’ lack of trust regarding how their personal data will be collected (eg, unknowingly through voice-activated devices) and handled (eg, by whom and how) [22,24-26,28,30,31,35,36,38,40,41,43,46]. However, 4% (1/26) of the studies reported no concerns regarding data sharing. This could be because of the respondents being chronic obstructive pulmonary disease patients who may have been used to their data being shared for clinical decision-making purposes [31]. Potential mitigation strategies suggested were to guarantee anonymity [26] and increase transparency in how the collected data will be used (eg, by which third party and how) [24,37]. There was a good proportion of representation from the general public, including patients (53.3%) [22,24-26,30,31,37,38,46] and health care providers and IT staff (Multimedia Appendix 3).

##### Patient Safety

Of the 26 studies, 21 (81%) discussed the respondents’ lack of trust in an AI to ensure patient safety while performing its tasks, especially regarding providing accurate information on rare conditions or unexpected situations [22-27,29-42,44]. Other concerns were regarding the credibility of AI-based recommendations (eg, whether it was validated by medical professionals) [30,32], maturity in the technology to provide safe and realistic recommendations [22,25], medical liability from the risk of medical errors and bias [26,35,36,44], secondary effects of AI-based diagnoses such as insurance claims [44], and miscommunications [26]. The potential mitigation strategies suggested were the provision of AI-specific regulations [30,31,41], transparency in its credibility, how a recommendation is derived (eg, showing who developed the system and the system reasoning and reliability based on information source and personal information), and its accuracy [32,38]. In contrast, 4% (1/26) of studies reported that the respondents were confident that the AI would outperform human clinical diagnoses because of higher accuracy and lower human errors [39]. Most respondents accepted AI in providing general health advice to minor ailments. Most of the responses represented the voices of the public, including patients (66.6%) [22-26,30-32,34,35,37-40] (Multimedia Appendix 3).

##### Technology

Of the 26 studies, 6 (23%) studies discussed the participants’ lack of trust in the maturity of AI technology in providing reliable and accurate information to support health-related predictions and recommendations [24,26,35,38,40,46]. This could be related to concerns over the lack of integration and synthesis of information from various sources, standardization of data collection, and the overall sustainability of AI-assisted health care service delivery [40,45]. However, 8% (2/26) of studies reported that respondents had similar trust in AI as compared with a human physician’s diagnoses [28,45]. Possible mitigation strategies include increasing system transparency and reporting system accuracies [26,46]. Only 8% (2/26) of studies represented the voices of health care and IT staff [35,40,49] (Multimedia Appendix 3).

#### Potential Impacts of Full Automation

In all, 46% (12/26) of studies discussed the perceptions of respondents on the possibility and impacts of full automation on the health care industry, especially in terms of diagnoses, all of which reported that it is unlikely that AI will completely replace health care professionals [22, 27, 29, 30, 33, 35, 36, 39, 42-44, 46]. This could largely be because of the immaturity of AI technology and its limitations in providing human-like interactions (which build trust) [27]. Instead, many patients preferred a combination of both AI and human physicians in diagnoses to achieve a more accurate and comprehensive evaluation [30,39]. Most of the responses represented the voices of health care and IT staff (58.3%) [27,29,35,36,42-44] (Multimedia Appendix 3).

### Needs to Improve Adoption of AI in Health Care

Besides the needs highlighted to mitigate the concerns, several additional features were found to potentially improve the adoption of AI in health care (Table 3).

#### Enhance Personalization and Customizability

Of the 26 studies, 6 (23%) studies discussed the need for AI to personalize information such as the explanation of diagnoses, recommendations, patient education, and even pertinent questions or issues to raise to their physicians [23,24,30-32,46]. Some studies also mentioned the need to customize chatbot features according to user preferences (for fixed options or free-texts) [23,24].

#### Enhance Empathy and Personification of AI-Enabled Chatbots and Avatars

In all, 27% (7/26) of studies highlighted the respondents’ concern over the lack of empathy, which is a crucial element of human interaction to build trust between service providers and consumers. However, empathy must be displayed tactfully in verbal and nonverbal expressions such that it does not appear to be “creepy and weird,” especially in populations with mental health issues [24]. Personification was also emphasized to increase the relatability, connection, and appeal to interact with the chatbot or avatar [23]. Perceived anonymity in interacting with the chatbot was also highlighted to assist in communication regarding sensitive topics [26].

#### Enhance User Experience, Design, and Interconnectedness With Other Devices

Overall, 15% (4/26) of studies described the need to improve user experience to increase user engagement with AI [23,25,28,31]. Strategies include needs-based interaction timing, the use of suitable verbal and nonverbal expressions, interconnectedness with other information sources (eg, electronic health record), apps (eg, calendar), and devices (eg, smart home technology–enabled devices).

#### Educate the Public on AI Capabilities

Of the 26 studies, 6 (23%) studies highlighted the lack of public and clinical awareness on the capabilities of AI in health care, of which the majority of the respondents expressed their willingness to learn [26,29,33,35,38,40]. A better understanding of the advantages and disadvantages of AI in health care could enhance the health care service delivery efficiency while balancing the expectations from it.

## Discussion

### Principal Findings

On the basis of the 26 articles included in this scoping review, we identified the perceptions and needs of various populations in the use of AI for general, primary, and community health care; chronic diseases self-management; self-diagnosis; mental health; and diagnostic procedures. However, the use of AI in health care remains challenged by the common perceptions, concerns, and unmet needs of various stakeholders such as patients, health care professionals, governmental or legal regulatory bodies, software developers, and industrial providers. Simply introducing AI into health care systems without understanding the needs of stakeholders will not lead to a sustainable change [50].

Our results showed that, similar to most ITs, AI was generally favored for its on-demand availability, ease of use, potential to improve efficiency, and reduce the cost of health care service delivery. These features could enhance patients’ compliance to health care treatments and recommendations that may be inaccessible or inconvenient. For example, patients are traditionally required to commit to a physician’s consultative appointment that could be relatively inflexible because of a long list of patients, and one could be forced to skip the consultation because of a conflict in their schedule. AI confers the benefit of information collection and dissemination beyond the constraints of time and place, which have been shown to improve medication adherence through an AI-based smartphone app [51] and diet and exercise adherence through an AI-based virtual health assistant [52]. Our findings also demonstrated that AI is valued for its potential to speed up health care processes such as diagnosis, waiting time, communication with care teams, decisional support, and other routine tasks (eg, progress monitoring) that can be automated. This increase in service delivery efficiency frees up time and resources for clinicians to focus on tasks that involve more unexpected variabilities such as dealing with rare disease management and interacting with patients, thereby reducing the risk of burnout, job dissatisfaction, and manpower shortage [53].

Although our findings showed high rates of acceptability, concerns were raised about the lack of trust (in data privacy, patient safety, and technology maturity) and the impacts of AI-driven automation on health care job security and health care services. Ethical controversies surrounding the use of AI in health care have been long-standing. Although there are increasingly more regulatory guidelines available, such as those developed by the World Health Organization [54] and the European Union [55], the use of AI in health care remains debatable because of the challenges in ensuring data privacy and proper data use [56]. This is especially true when data collection modes are conducted through third-party apps, such as Facebook Messenger (Meta Platforms), of which privacy policies are governed by technology companies and not health care institutions [24]. Moreover, although there are privacy and security precautionary measures, the increasing reports of data leaks and vulnerabilities in electronic medical record databases erode population trust. Future security and transparency measures could consider the use of blockchain technology, and privacy laws should be properly delineated and transparent [57].

This review also found the need to enhance the personalization and customizability of information provided by AI, the incorporation of empathy and personification in AI-based conversational agents, the user experience through better design and interconnectedness with other devices and systems, and the need to educate the public on AI capabilities. Concerning personalized health care, reports generated by AI should be integrated and explained in accordance with each individual’s demographic and clinical profile to facilitate self-management [46]. We also identified the need for AI to not only assist in the understanding of patients’ medical condition but also the provision of relevant treatment options and personalized recommendations with intuitive actions provided (eg, a button to call an ambulance when deemed necessary by the AI) [31]. This coincides with existing studies that highlight the predictive power of AI in providing support to preventive disease onset or deterioration through interventions tailored according to user preferences [58]. For example, AI has been used to provide just-in-time adaptive interventions that prompt users to perform healthy behavior changes (eg, healthy diet and exercise and smoking cessation) based on constant data collection of their behaviors and preferences [49]. However, the data collection of users’ behavioral or clinical information should also consider the customizability of input options (eg, providing predefined options or allowing for free-text input) to enhance the usability and adoption of such systems, depending on user preferences [24]. Personification of AI-based conversational agents to express human-like identity, personality, empathy, and emotions was also highlighted as an area of improvement to enhance human-chatbot interactions and eventually user adoption [59]. It was also important for the AI systems to be accessible through various devices (eg, tablets, televisions, laptops, and smart home appliances) and modes (eg, text and speech) for the convenience of information consumption and data collection. Finally, our findings suggest a need to address the knowledge deficit in the definition, capacity, and functions of AI. This could be done by cultivating AI literacy and exposure from childhood [60] and incorporating the AI curriculum in health care training and upgrading courses [61].

Overall, our study findings are consistent with well-established theories such as the Technology Acceptance Model, of which the second version proposed by Venkatesh and Davis [62] posits that technology acceptance is strongly associated with the perceived usefulness and perceived ease of use, which are influenced by subjective norms, images, job relevance, output quality, result demonstrability, experience, and voluntariness [63]. Therefore, to enhance the acceptability of AI in health care applications, its perceived usefulness over and above the current standard practices such as capacity to increase service delivery efficiency and community-based self-diagnostic accuracy should be emphasized. Such messages should be designed to be relevant to the individual and organizational adopters of a social system through various communication channels and change agents (ie, gatekeepers and opinion leaders). Such messages should be persuasive to spark five stages of adoption, namely, knowledge, persuasion, decision, implementation, and confirmation, known as the diffusion of innovation theory by Rogers [64]. Different strategies are also needed to correspond with the different categories of adopters, namely, the innovators, early adopters, early majority, late majority, and laggards. Different rates of technology adoption are associated with one’s risk tolerance related to higher social economic status, education level, and financial stability [65]. An example is the case of AI adoption in chronic disease early detection and management in the United Arab Emirates. Success was attributed to the *managerial, organizational, operational, and IT infrastructure factors* that contribute to the factors of the Technology Acceptance Model [66]. However, advanced technologies such as AI continue to be relatively expensive and require eHealth literacy, which may widen the digital divide, and therefore the data divide and health disparity among societies. According to a report published in *The Lancet*, the internet remains inaccessible to approximately 50% of the global population because of a digital divide [67]. In addition, there are specific guidelines on the implementation of AI in health care service delivery, such as the quality of data and certification of AI systems, which may deter adoption [68].

### Limitations

This study had several limitations. First, only articles written in English were retrieved, possibly limiting the comprehensiveness of our findings. However, we conducted a search on Google Scholar to supplement the electronic database search for more relevant papers. Second, the studies were largely heterogeneous in their study designs, research aims, and data collection methods. Third, there were limited studies on the perceptions of AI and clinical researchers who could provide outlooks on the perceptions of the general public. Finally, the public’s perceptions of AI in health care may be limited by their knowledge of the definitions and capabilities of AI, as highlighted in our findings that there is a need to enhance the public’s knowledge on AI. Therefore, the priority or importance of each perception and need could not be evaluated. The inclusion of articles based on our definition of AI could also have limited the scope of this study. Studies that considered different definitions of AI may have been excluded.

### Recommendations for Future Design and Research

This study highlighted the perceptions and needs of AI to enhance its adoption in health care. However, one major challenge lies in the extent to which AI is tailored according to each individual’s unique preference, and if such preferences are largely varied, how data can be aggregated for analyses and applicability in specific health care applications. Therefore, future studies that use AI should not only consider the issues raised in this study but also clarify the applicability in their applications and target population. A prior needs-based analysis is recommended before the development of AI systems.

### Conclusions

Although AI is valued for its 24/7 availability in health care service delivery, ease of use, and capacity to improve health care service provision efficiency, concerns over trust in data privacy, information credibility, and technological maturity remain. Although several mitigation strategies such as enhancing transparency over predictive accuracy and information sources were identified, other areas of improvement were also highlighted. Future studies and AI development should consider the points raised in this study to enhance the adoption and enhancement of AI to improve health care service delivery.

## Appendix

Multimedia Appendix 1 PRISMA (Preferred Reporting Items for Systematic Reviews and Meta-Analyses) checklist.

Multimedia Appendix 2 Database search details.

Multimedia Appendix 3 Study characteristics.

Multimedia Appendix 4 Acceptability of artificial intelligence use in health care.

## Abbreviations

AGI: artificial general intelligence
AI: artificial intelligence
ANI: artificial narrow intelligence
IT: information technology
PRISMA: Preferred Reporting Items for Systematic Reviews and Meta-Analyses
