# Supplementary material for: Perceptions and Needs of Artificial Intelligence in Health Care to Increase Adoption: Scoping Review
Source: J Med Internet Res. 2022 Jan 14;24(1):e32939. doi: 10.2196/32939 (PMC8800095; doi:10.2196/32939)
Supplement: Multimedia Appendix 2 [file jmir_v24i1e32939_app2.docx]

**Appendix 2:** Database search details.

| Topic | Search terms | Number of studies |
| --- | --- | --- |
| ACM Library | [[Abstract: "artificial intelligence"] OR [Abstract: or "ai"]] AND [Abstract: public or consumer or community] AND [Abstract: perception* or preference* or needs* or opinions* or acceptability] | 130 |
| CINAHL | AB ( "artificial intelligence" or "AI" ) AND AB ( public or consumer or community ) AND AB ( perception* OR preference* OR needs* OR opinions* OR acceptability) | 142 |
| Embase | '('artificial intelligence':ab,ti OR 'ai':ab,ti) AND (public:ab,ti OR consumer:ab,ti OR 'community':ab,ti) AND (perception*:ab,ti OR preference*:ab,ti OR needs*:ab,ti OR opinions*:ab,ti OR acceptability:ab,ti) | 327 |
| IEEE Xplore | ("Abstract":"artificial intelligence" OR "Abstract":"AI") AND ("Abstract":public OR "Abstract":consumer OR "Abstract":community) AND ("Abstract":perception* OR "Abstract":preference* OR "Abstract":needs* OR "Abstract":opinions* OR "Abstract":acceptability) | 169 |
| PsycINFO | (("artificial intelligence" or "AI") and (public or consumer or community) and (perception* or preference* or needs* or opinions* or acceptability)).ab. | 146 |
| PubMed | (("artificial intelligence"[Title/Abstract] OR AI[Title/Abstract] ) AND (public [Title/Abstract] OR consumer [Title/Abstract] OR community[Title/Abstract]) ) AND (perception*[Title/Abstract] OR preference*[Title/Abstract] OR needs*[Title/Abstract] OR opinions*[Title/Abstract] OR acceptability*[Title/Abstract]) | 295 |
| Scopus | ( ABS ( "artificial intelligence" OR "AI" ) AND ABS ( public OR consumer OR community ) AND ABS ( perception* OR preference* OR needs* OR opinions* OR acceptability ) ) | 1,310 |
| The Cochrane Library | "artificial intelligence" or "AI" in Title Abstract Keyword AND public or consumer or community in Title Abstract Keyword AND perception* OR preference* OR needs* OR opinions* OR acceptability in Title Abstract Keyword - (Word variations have been searched) | 83 |
| Web of Science | TOPIC: ("artificial intelligence" or "AI") AND TOPIC: (public or consumer or community) AND TOPIC: (perception* OR preference* OR needs* OR opinions* OR acceptability)  Timespan: All years. Indexes: SCI-EXPANDED, SSCI, A&HCI, CPCI-S, CPCI-SSH, ESCI. | 1,064 |
| Google Scholar (first 10 search-result pages) | "artificial intelligence" AND (public opinions perceptions needs applications) | 6 |
|  | Total | 3,666 |
