# Supplementary material for: Perceptions and Needs of Artificial Intelligence in Health Care to Increase Adoption: Scoping Review
Source: J Med Internet Res. 2022 Jan 14;24(1):e32939. doi: 10.2196/32939 (PMC8800095; doi:10.2196/32939)
Supplement: Multimedia Appendix 3 [file jmir_v24i1e32939_app3.docx]

**Appendix 3**: Study characteristics.

| **Author, year** | **Aim** | **Study design** | **Country** | **AI applications in healthcare** | **Data collection method** | **Population characteristics** | **N** | **Mean age** | **% Male** |
| --- | --- | --- | --- | --- | --- | --- | --- | --- | --- |
| Abdi et al., 2021 | To establish a consensus of opinions on the potential of 10 emerging technologies to meet older people’s needs in five care and support domains. | Observational | UK, Cyprus, Australia, Netherlands, Sweden, Spain, US, Canada | Older people’s community healthcare needs | Two-round Delphi survey | Healthcare and technology staff | 21 | nil | 48 |
| Abdullah et al., 2020 | To explore health care employees’ perceptions and attitudes toward the implementation of artificial intelligence technologies in health care institutions in Saudi Arabia. | Observational | Saudi Arabia | General healthcare | Online questionnaire | Healthcare and technology staff | 250 | 74.8% 20-40 yo | 25.2 |
| Baldauf et al., 2020 | To present the results on user perceptions of AI-driven self-diagnosis apps. | Observational | Switzerland | Self-diagnosis | Online questionnaire | General public | 106 | 34 | 58.5 |
| Castagno et al., 2020 | To investigate the prior knowledge of health professionals on the subject of AI as well as their attitudes and worries about its current and future applications. | Observational | UK | General healthcare | Online questionnaire | Healthcare staff | 98 | NS | NS |
| Easton et al., 2019 | To co-design the content, functionality, and interface modalities of an autonomous virtual agent to support self-management for patients with an exemplar long-term condition (LTC; chronic pulmonary obstructive disease [COPD]) and then to assess the acceptability and system content. | Mixed-method | UK | COPD self-management | Interview & questionnaire | COPD patients and their caregivers | 11 | workshop 1: 82.5, 69-86 workshop 2: 73, 66-80 | NS |
| Gao et al., 2020 | To explore the public perception of AI in medical care through a content analysis of social media data, including specific topics that the public is concerned about; public attitudes toward AI in medical care and the reasons for them; and public opinion on whether AI can replace human doctors. | Observational | China | General healthcare | Sina Weibo platform public posts from January to December 2017. | Sina Weibo users | 1764 posts | 30 | 73.4 |
| Griffin et al., 2021 | To better understand patient information needs and perceptions of chatbots to support hypertension medication self-management. | Mixed-method | US | Hypertension self-management | Interview & questionnaire | Hypertension patients | 15 | 59 | 59 |
| Kim et al., 2019 | To analyze the subjects to understand dementia patients’ level of need for aid tools that affect their daily activities, safety management, and cognitive function. | Observational | Korea | Cognitive aid for dementia | Questionnaires | Normal elderly, thirteen dementia patients, and fifteen caregivers of dementia | 57 | 72.6 | 37.6 |
| Lai et al., 2020 | To provide an overview of how health professionals perceive the arrival of AI in their practices and what influences their views. | Qualitative | France | General healthcare | interview | Healthcare and industrial staff | 40 | NS | NS |
| Li et al., 2020 | To study the public's cognition of the intelligent self-diagnosis system, and discusses how to effectively improve the user's recognition and trust of the intelligent self-diagnosis system. | Mixed-method | China | Self-diagnosis | Interview & questionnaire | General public | 13 | 35 | 80% |
| Liu et al., 2021 | To quantify and compare people’s preferences for AI clinicians and traditional clinicians before and during the COVID-19 pandemic, and to assess whether people’s preferences were affected by the pressure of pandemic. | Observational | China | Self-diagnosis | Online questionnaire | General public | 528 | 68.85 | 48.5 |
| Liu et al., 2021 | To measure the extent of patients’ preferences for a range of characteristics of an AI diagnosis scheme in China and to determine what characteristics are more attractive and make AI a better alternative to defeat traditional medical methods. | Observational | China | Self-diagnosis | Online questionnaire | Outpatients | 767 | 18-85 | 48.1 |
| Liyanage et al., 2019 | To form consensus about perceptions, issues, and challenges of AI in primary care. | Observational | UK | Primary healthcare | Three-round Delphi | Healthcare and technology staff | 20 | NS | NS |
| McCradden et al., 2020 | To understand the perspectives of the general public regarding the use of health data in AI research. | Qualitative | Canada | General healthcare | Interview | General public | 41 | 40 | 51.2 |
| McCradden et al., 2020 | To investigate current perspectives on ethical issues surrounding AI in health care | Qualitative | Canada | General healthcare | Interview | healthcare providers and meningioma patients | 30 | 51.6 | 30 |
| Milne-Ives et al., 2020 | To assess the effectiveness and usability of conversational agents in health care and identify the elements that users like and dislike to inform future research and development of these agents. | Systematic review | UK | General healthcare | Literature review | NA | 31 articles | NS | NS |
| Nadarzynski et al., 2019 | To explore participants’ willingness to engage with AI-led health chatbots. | Mixed-method | UK | General healthcare | Interview & questionnaire | university students | 29 | 30 | 39 |
| Okolo et al., 2021 | To explore community health workers' perceptions of an AI application for automated disease diagnosis. | Qualitative | India | Community healthcare | Interviews | Healthcare staff | 21 | 44 | 0 |
| Palanica et al., 2019 | To investigate the perceptions of physicians regarding the use of health care chatbots, including their benefits, challenges, and risks to patients. | Observational | Canada | General healthcare | Online questionnaire | Healthcare staff | 100 | 44.9 | 69 |
| Prakash et al., 2020 | To understand the factors influencing the adoption and use of Intelligent conversational agents (Cas) in mental healthcare by examining the perceptions of actual users. | Secondary | India | Mental healthcare | User reviews of popular mental health chatbot apps on the app stores | App users | 1,826 reviews | NS | NS |
| Scheetz et al., 2021 | To ascertain their current use, understanding and perceptions of AI. | Observational | Australia, New Zealand | Ophthalmology, radiology/radiation oncology, dermatology | Online questionnaire | Healthcare staff | 632 | NS | NS |
| Stai et al., 2020 | To understand better the public perception and comprehension of medical technology such as AI and robotic surgery. | Observational | US | Diagnostics and robotic surgery | Questionnaires | General public | 264 | 45 | 42 |
| Sun et al., 2019 | To map the challenges in the adoption of AI in the public sector as perceived by key stakeholders. | Mixed-method (primary + secondary data) | China | General healthcare | Primary interviews and secondary government policy documents, excerpts | Government, healthcare and technology staff | 19 | NS | NS |
| Tam-Seto et al., 2020 | To evaluate the perceptions of the use of an AI-supported mental health app by the Canadian military community. | Qualitative | Canada | Mental health | Interviews | Military individuals, family members, veterans, health care providers | 44 | NS | NS |
| Xiang et al., 2020 | To investigate public perceptions, receptivity, and demands regarding the implementation of medical AI. | Observational | China | General healthcare | Online questionnaire | General public | 2780 | 30-39 (40.72) | 45.36 |
| Zhang et al., 2021 | To understand patients’ perceptions and acceptance of using AI technology to interpret their radiology reports | Observational | US | Radiology imaging data | Interviews | General patients | 13 | 18-64 | 46.2 |

Note: AI=Artificial intelligence; IRJ paper=Internationally-referred Journal; NS=Not specified
