# Supplementary material for: Perceptions and Needs of Artificial Intelligence in Health Care to Increase Adoption: Scoping Review
Source: J Med Internet Res. 2022 Jan 14;24(1):e32939. doi: 10.2196/32939 (PMC8800095; doi:10.2196/32939)
Supplement: Multimedia Appendix 4 [file jmir_v24i1e32939_app4.docx]

**Appendix 4:** Acceptability of AI use in healthcare.

| **Author, year** | **Acceptability measure** | **Acceptability level** |
| --- | --- | --- |
| Abdi et al., 2021 | Median, interquartile range (IQR) and frequency distribution of Delphi survey | 70% agrees upon score of 4 or 5; or disagrees upon score of 1 or 2 |
| Abdullah et al., 2020 | Modified version of an existing questionnaire | Moderate level of acceptance |
| Baldauf et al., 2020 | Self-developed questions on willingness to use, trust, interplay with physician, expected features and subjective pros and cons | mean score for each item was 3-4 (5-point rating scale) |
| Castagno et al., 2020 | NS | NS |
| Easton et al., 2019 | System Usability Scale (SUS) questionnaires | Median rating 73.75/100. 50% of the participants strongly agreed with the statement “I think that I would like to use this system frequently,” and the other 50% (4/8) were unsure. |
| Gao et al., 2020 | NS | NS |
| Griffin et al., 2021 | Unified theory of acceptance and use of technology (UTAUT) | Age is a moderator of one’s acceptance and behavioral intention to use a technology. |
| Kim et al., 2019 | NS | NS |
| Lai et al., 2020 | NS | NS |
| Li et al., 2020 | Interview | Most of the volunteers expressed their willingness to use intelligent self-diagnosis system. |
| Liu et al., 2021a | NS | NS |
| Liu et al., 2021b | Discrete choice experiment (DCE) MCQ | 55.8% of the respondents opted for AI diagnosis regardless of the description of the clinicians. |
| Liyanage et al., 2019 | NS | NS |
| McCradden et al., 2020a | NS | NS |
| McCradden et al., 2020b | NS | NS |
| Milne-Ives et al., 2020 | NS | NS |
| Nadarzynski et al., 2019 | one question: ‘How likely would you be to use a health chatbot in the next 12 months if it was available to you today?’ with five options (from ‘extremely unlikely’ to ‘extremely likely’) | Majority of participants were willing to use chatbots for minor health concerns that would not require a physical examination.  Moderate acceptability (67%), correlated negatively with perceived poorer IT skills and dislike for talking to computers as well as positively correlated with perceived utility, positive attitude and perceived trustworthiness. |
| Okolo et al., 2021 | NS | NS |
| Palanica et al., 2019 | NS | NS |
| Prakash et al., 2020 | NS | NS |
